# Targeting Paretic Propulsion and Walking Speed With a Soft Robotic Exosuit: A *Consideration-of-Concept* Trial

**DOI:** 10.3389/fnbot.2021.689577

**Published:** 2021-07-28

**Authors:** Franchino Porciuncula, Teresa C. Baker, Dheepak Arumukhom Revi, Jaehyun Bae, Regina Sloutsky, Terry D. Ellis, Conor J. Walsh, Louis N. Awad

**Affiliations:** ^1^Paulson School of Engineering and Applied Sciences, Wyss Institute for Biologically Inspired Engineering, Harvard University, Cambridge, MA, United States; ^2^Neuromotor Recovery Laboratory, Department of Physical Therapy, College of Health and Rehabilitation Sciences, Sargent College, Boston University, Boston, MA, United States; ^3^Apple Inc., Cupertino, CA, United States

**Keywords:** stroke, exosuit, walking, gait training, neurorehabilitation, propulsion, wearable robots, soft robotics

## Abstract

**Background:** Soft robotic exosuits can facilitate immediate increases in short- and long-distance walking speeds in people with post-stroke hemiparesis. We sought to assess the feasibility and rehabilitative potential of applying propulsion-augmenting exosuits as part of an individualized and progressive training program to retrain faster walking and the underlying propulsive strategy.

**Methods:** A 54-yr old male with chronic hemiparesis completed five daily sessions of Robotic Exosuit Augmented Locomotion (REAL) gait training. REAL training consists of high-intensity, task-specific, and progressively challenging walking practice augmented by a soft robotic exosuit and is designed to facilitate faster walking by way of increased paretic propulsion. Repeated baseline assessments of comfortable walking speed over a 2-year period provided a stable baseline from which the effects of REAL training could be elucidated. Additional outcomes included paretic propulsion, maximum walking speed, and 6-minute walk test distance.

**Results:** Comfortable walking speed was stable at 0.96 m/s prior to training and increased by 0.30 m/s after training. Clinically meaningful increases in maximum walking speed (Δ: 0.30 m/s) and 6-minute walk test distance (Δ: 59 m) were similarly observed. Improvements in paretic peak propulsion (Δ: 2.80 %BW), propulsive power (Δ: 0.41 W/kg), and trailing limb angle (Δ: 6.2 degrees) were observed at comfortable walking speed (*p*'s < 0.05). Likewise, improvements in paretic peak propulsion (Δ: 4.63 %BW) and trailing limb angle (Δ: 4.30 degrees) were observed at maximum walking speed (*p*'s < 0.05).

**Conclusions:** The REAL training program is feasible to implement after stroke and capable of facilitating rapid and meaningful improvements in paretic propulsion, walking speed, and walking distance.

## Introduction

In healthy walking, the anteriorly-directed ground reaction forces generated by each limb act to propel the body forward (Shumway-Cook and Woollacott, 2001; Bowden et al., 2006; Kuo and Donelan, 2010). In post-stroke hemiparetic walking, impaired propulsion by the paretic limb (Bowden et al., 2006; Awad et al., 2015a) hinders the forward acceleration of the body (Kuo and Donelan, 2010), resulting in an increased energy cost of walking (Penke et al., 2019) and slower walking speeds (Bowden et al., 2006; Awad et al., 2015a). As walking speed is a key indicator of functional independence (Fulk et al., 2017), improving walking speed is a top priority during post-stroke rehabilitation (Bohannon et al., 1988); however, compensations within and between limbs may allow functional walking speeds despite persisting impairments in paretic propulsion (Bowden et al., 2006; Cruz et al., 2009). That is, focusing only on increasing walking speed during gait training is not sufficient to facilitate paretic limb neuromotor recovery (Roelker et al., 2019); the benefits of faster walking may be overshadowed by persisting metabolically-expensive and unstable gait patterns (Mahon et al., 2015; Vistamehr et al., 2016; Balbinot et al., 2020).

**Figure 1:**
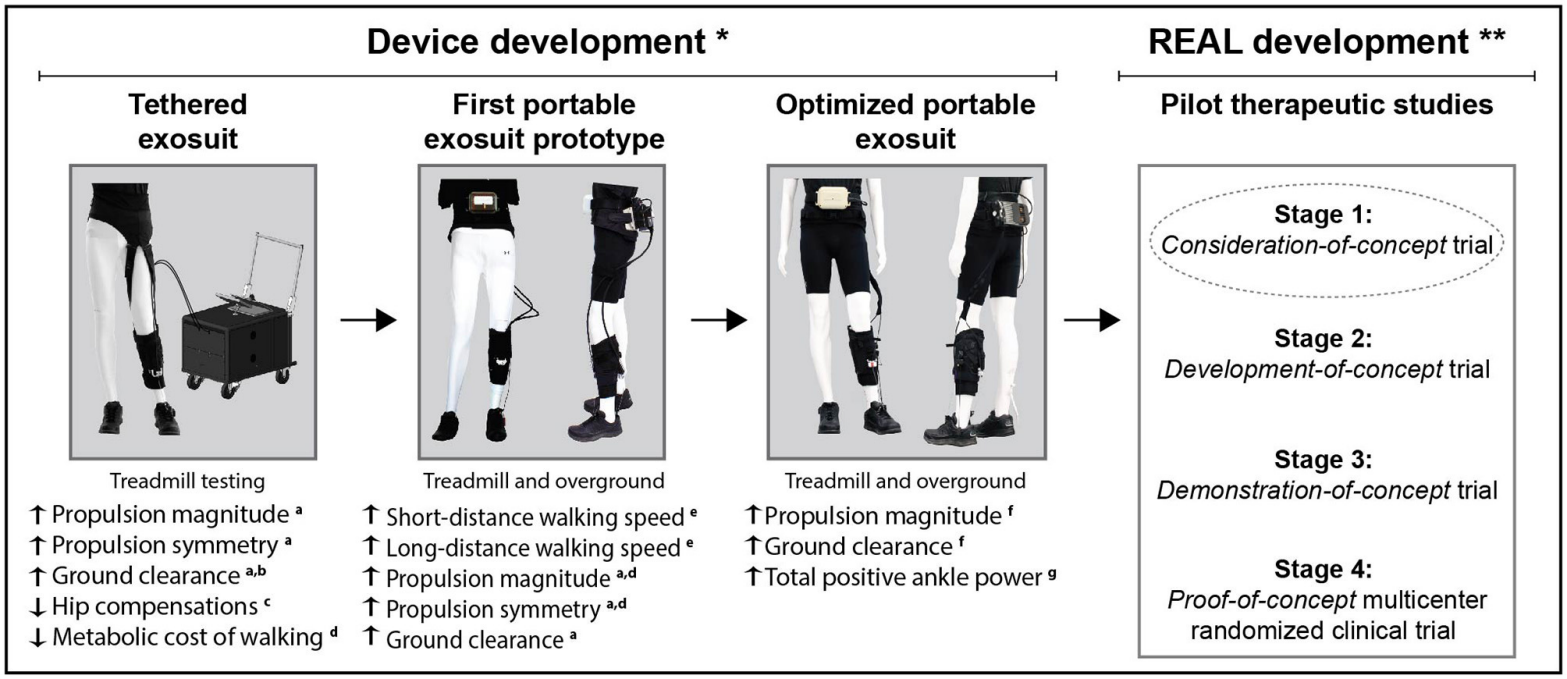
Progression of our stroke exosuits program from device development to development of the Robotic Exosuit Augmented Locomotion (REAL) gait training program. a, Awad et al. (2017a); b, Bae et al. (2015); c, Awad et al. (2017b); d, Bae et al. (2018b); e, Awad et al. (2020a); f, Bae et al. (2018a); g, Siviy et al. (2020). *Single-session comparisons of exosuit powered vs. unpowered (or not worn); **Multi-session evaluations of pre-to-post training effects of REAL gait training.

Next-generation soft wearable robots, called exosuits, assist paretic dorsiflexion during swing phase to facilitate ground clearance and paretic plantarflexion during stance phase to enhance propulsion (Bae et al., 2015, 2018a). Preliminary research on exosuits that focused on device development (Bae et al., 2015, 2018a; Awad et al., 2017a,b) (Figure 1) demonstrated immediate, within-session improvements in paretic ground clearance and forward propulsion (Awad et al., 2017a), inter-limb symmetry (Awad et al., 2017a; Bae et al., 2018a), walking economy (Bae et al., 2018b), and reduced gait compensations (Awad et al., 2017b). Together, these immediate biomechanical benefits enabled clinically-meaningful increases in both short- and long-distance walking speeds (Awad et al., 2020a). Though promising, the value of exosuits in the context of gait rehabilitation is not clear; the potential for training-related effects that are retained beyond the use of exosuits are not known.

The field of rehabilitation robotics has advanced on the promise of facilitating massed stepping practice with ease (Esquenazi et al., 2017; Hobbs and Artemiadis, 2020); however, the collective evidence falls short in showing therapeutic benefits that exceed those of conventional approaches (Tedla et al., 2019; Hornby et al., 2020). A criticism of previous clinical trials has been the sub-optimal staging of preliminary studies to shape the robotic interventions that were ultimately tested in large multi-center trials. That is, before progressing to large trials designed to test clinical efficacy, there is value in systematically staging pilot trials to develop, refine, and validate the robotic intervention's theoretical bases (Dobkin, 2009). As a first step in the progressive staging of clinical trials, we undertook a single-subject *consideration-of-concept* trial (Dobkin, 2009) to inform the development of a propulsion-targeting exosuit-augmented gait training program, with a focus on the optimization of its outcomes and design elements for subsequent clinical trials.

Synthesizing our team's previous findings on the immediate effects of soft robotic exosuits on post-stroke walking (Bae et al., 2015, 2018a; Awad et al., 2017a,b), we designed the Robotic Exosuit Augmented Locomotion (REAL) gait training program. REAL training leverages the soft robotic exosuit's ability to immediately enhance paretic propulsion and walking speed, together with individualized and progressive speed training and goal-based strategic feedback, to therapeutically train faster walking by way of a more typical propulsive strategy. The aims of this study were (1) to design the REAL gait training program and examine its feasibility on a single subject with chronic hemiparesis and (2) to evaluate its rehabilitative potential to improve both walking speed and propulsion function. Building on our previous findings of immediate improvements in speed and propulsion when walking *with* a soft robotic exosuit (Awad et al., 2017a, 2020a), we hypothesized that REAL training would facilitate therapeutic improvements in speed and propulsion measured *without* the exosuit.

## Methods

### Robotic Exosuit Augmented Locomotion Gait Training

**Figure 2:**
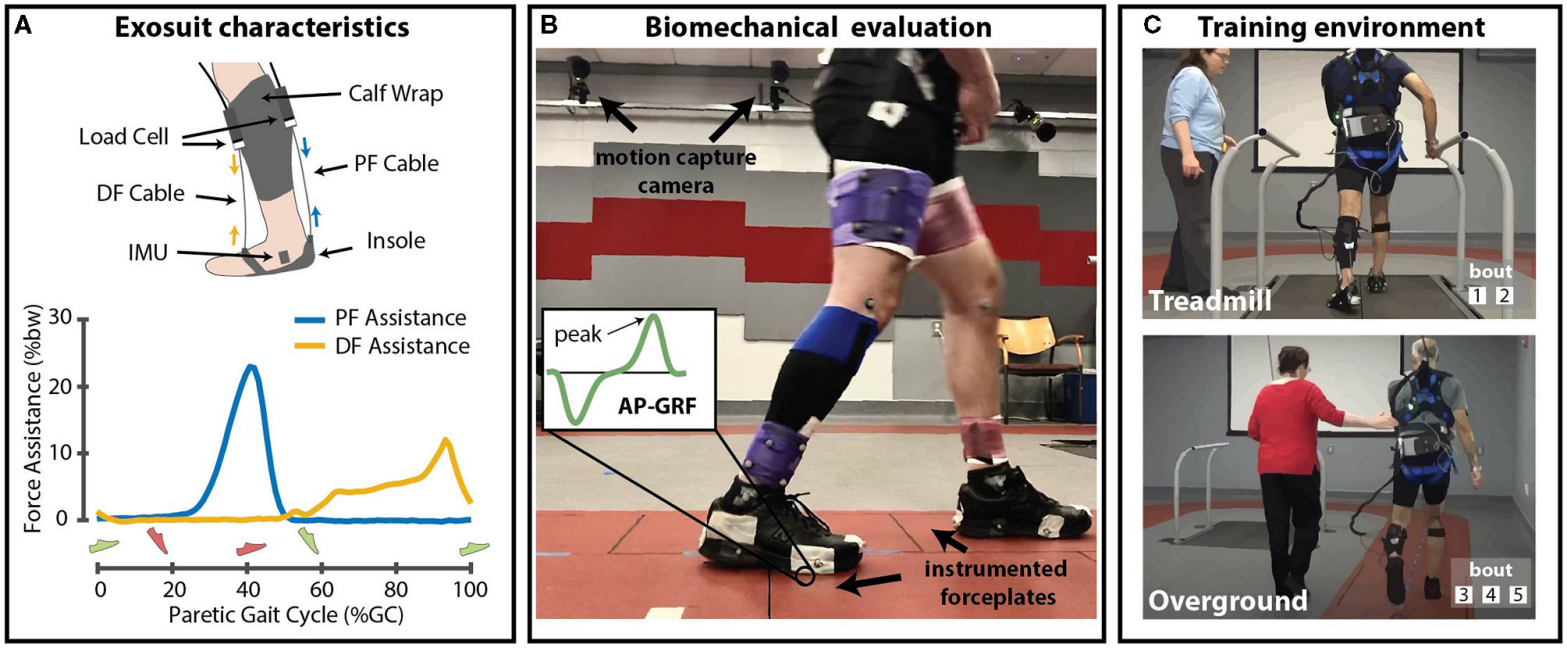
**(A)** Schematic of soft robotic exosuit with representative force profiles for dorsiflexion and plantarflexion assistance. **(B)** Laboratory setup for biomechanical evaluation of overground walking. **(C)** Illustration of Robotic Exosuit Augmented Locomotion (REAL) training, administered by a physical therapist on the treadmill and overground. DF, Dorsiflexion; PF, Plantarflexion; AP-GRF, Anterior-posterior ground reaction force; %bw, Percent body weight; IMU, Inertial measurement unit. **(A)** Green shoe refers to paretic foot, and red shoe refers to non-paretic foot.

REAL gait training, as tested in this initial pilot, consisted of five daily sessions. Each session consisted of 30 min of total walking practice divided into five 6-min training bouts. The first two bouts were conducted on the treadmill, followed by three bouts overground (Figure 2C). The maximum allowable heart rate at each training visit was 85% of the peak heart rate observed during an electrocardiography-monitored graded exercise test completed prior to training.

The task selection for the REAL gait training program was based on high intensity, task-specific, and progressively challenging walking practice—principles which are known to be important in motor learning (Guadagnoli and Lee, 2004; Kleim and Jones, 2008) and relevant for contemporary robot-augmented rehabilitation interventions (Morone et al., 2017). To operationalize these principles, REAL implements the following design elements:

#### High Intensity

Training at higher practice intensities is a more potent approach to improving walking competency post-stroke than low to moderate intensity exercises (Luo et al., 2019). In the REAL training program, high-intensity training is implemented through a speed-based approach; faster speeds are explicitly encouraged during training via real-time feedback and supported by the exosuit's ability to enable faster and farther walking (Awad et al., 2020a). This approach is consistent with recent clinical practice guidelines that promote fast walking as a means to increase practice intensity for stroke rehabilitation (Hornby et al., 2020). For each training bout, the participant is directed to walk at or faster than a target training speed (*Speed*_*Target*_), computed as follows:

(1) $$\begin{array}{l} Speed_{Target}=CWS+0.5\;\left(MWS-CWS\right) \end{array}$$

with CWS and MWS being the participant's comfortable and maximum walking speeds measured daily from a 10-meter Walk Test (10mWT). Moreover, to maximize training intensity for this *consideration-of-concept* trial, REAL training was administered every day for five consecutive days.

#### Task-Specificity

Task-specificity is implemented both at the level of function and impairment. First, the entirety of the REAL training program is based on the direct practice of the task of walking. Second, REAL is designed to target specific gait impairments *during* walking practice—an approach that has been shown to be more effective than interventions that target impairments isolated from the task of walking (Nadeau et al., 2013; Forrester et al., 2016). More specifically, exosuit-augmented walking practice is combined with feedback from a licensed physical therapist, with both aimed at facilitating fast walking speeds through a propulsion-based strategy. That is, throughout each session, the participant is actively engaged in identifying gait pattern goals related to increasing speed and propulsion, with the physical therapist providing summary feedback on how well the target training speeds are achieved. In addition, the physical therapist provides strategies designed to increase forward propulsion (e.g., pushing off the ground, increasing plantarflexion or the trailing limb angle, or modulating the non-paretic step length).

#### Progressively Challenging

Progressively challenging practice conditions (Guadagnoli and Lee, 2004) are posited to enhance motor learning. In the REAL training program, progression of training complexity is achieved through the progression of *Speed*_*Target*_ and the staged introduction of an intermittent assistance schedule. More specifically, when *Speed*_*Target*_ is regularly achieved, the REAL gait training program allows for progression by transitioning from continuous to intermittent exosuit assistance. Intermittent exosuit assistance consists of repeated serial switching of the exosuit power on (1.5 min) and off (0.5 min) during each training bout. When the exosuit is powered off, the participant is encouraged to replicate their gait pattern from when the exosuit was powered on. The alternating assistance schedule is posited to enhance motor skill transfer (Müssgens and Ullén, 2015).

### History and Examination

The study participant was a 54-year old male with chronic left-sided hemiparesis after a right middle cerebral artery ischemic stroke, status-post patent foramen ovale surgery, 5.6 years prior to enrollment. The participant reported that he routinely walked with a cane and foot-up brace and had limited ability to walk without these devices. This was confirmed at the initial clinical assessment where the Fugl-Meyer Assessment was also completed, yielding a Lower Extremity motor sub-score of 24/34, and lower extremity sensation sub-score of 12/12. Baseline assessments of postural stability during walking and general mobility were assessed through the Functional Gait Assessment (16/20) and the Timed Up-and-Go (TUG) test (14.47 s), which were both tested without an assistive device or brace. The participant was not receiving any physical therapy at the time of the intervention.

### Design

A single-subject *consideration-of-concept* trial with repeated baseline measurements assessed the feasibility and rehabilitative potential of REAL. Enrollment in this study was based on convenience sampling. The study participant was known to the research team through his participation in 44 research visits over a 2-year period during the development phase of our exosuits program. Because gait training was not provided at these visits, the repeated measurements during this period provided a robust baseline assessment of walking speed prior to his participation in REAL training. In addition to the repeated baseline measurements made over this 2-year period, a dedicated pre-training baseline evaluation was completed prior to the five daily sessions of REAL training. A post-training evaluation was completed after. The study was approved by the Institutional Review Boards at Harvard University and Boston University. The funders played no role in the design, conduct, or reporting of the study.

### Exosuit

The soft robotic exosuit used in this study is detailed in previous work (Bae et al., 2018a; Siviy et al., 2020). Briefly, the exosuit consists of functional apparel that is worn on the paretic leg. A shoe insole and calf wrap provide textile attachment points for Bowden cables located in front of and behind the paretic ankle. An actuator unit and battery are secured close to the body center-of-mass using a waist belt. From the actuation unit, Bowden cables connect to the attachment points on the calf wrap and insole. Retraction of the Bowden cables deliver assistive dorsiflexor and plantarflexor torques during targeted phases of the gait cycle. Shoe-mounted inertial sensors enable gait detection and the delivery of the assistive forces in synchrony with the wearer's gait.

An admittance control approach was used for plantarflexor assistance where an explicit force profile was commanded (Figure 2A), with the maximum force set at 180N [i.e., 25 %bodyweight (%bw)], with onset time at 38–41% of the gait cycle (Siviy et al., 2020). Conversely, dorsiflexor assistance used a position controller, with the assistance parameters determined by a physical therapist through visual gait observation (Bae et al., 2018a). Though this force profile was tuned during the first study visit, it was not allowed to vary over time (i.e., commanded force profile parameters were fixed within and across days).

### Measures and Data Analysis

To examine the therapeutic effects of REAL training, all clinical and biomechanical assessments were tested overground without wearing the exosuit. No assistive device or brace was used in all assessments.

#### Clinical Outcomes

To establish a stable baseline, data from five comfortable walking speed 10-meter walk tests (10mWT) conducted over a 2-year period were used. Four of these measurements were taken from historical data obtained during the development phase of this project, and the fifth measurement was collected at the pre-training evaluation that immediately preceded the training period. Additionally, to fully characterize short- and long-distance walking function, maximum walking speed during the 10mWT and 6-Minute Walk Test (6MWT) distance were measured at both the pre- and post-training evaluations. The 10mWT comfortable walking speed served as our main clinical outcome, whereas the 10mWT maximum walking speeds and 6MWT distance were secondary outcomes.

#### Biomechanical Measurements and Outcomes

Locomotor biomechanics were collected during the pre and post-training 10mWTs using an 18-camera motion capture system (Oqus, Qualysis, Gottenburg, Sweden; 200 Hz) and six instrumented forceplates (Bertec, Columbus, Ohio, USA; 2000Hz) (Figure 2B). Kinematic and kinetic data were filtered using a zero-lag low-pass 4th order Butterworth filter with 10 Hz cutoff frequency. Joint angles were computed using direct kinematics and joint moments and powers using inverse dynamics (Visual 3D, C-Motion, Germantown, MD USA). All biomechanical data were time-synchronized and normalized between sequential heel strikes measured using a 30N vertical ground reaction force threshold.

Primary biomechanical outcomes included measurements of paretic propulsion (i.e., peak, impulse, and symmetry of the anterior ground reaction forces) and related metrics (Hsiao et al., 2015, 2016b; Fickey et al., 2018) such as plantarflexor moment, plantarflexor power, ankle angular velocity, and trailing limb angle. Secondary measures included gait quality metrics such as stride length, cadence, hip hiking, circumduction, and braking force (i.e., peak, impulse, and symmetry of the posterior ground reaction forces). Percentage of symmetry of propulsion and braking forces were calculated by dividing the value of the paretic limb by the sum of paretic and non-paretic limbs.

#### Statistical and Clinical Analyses

To establish a baseline comfortable walking speed, the Kruskal-Wallis test compared the five repeated baseline measurements. To examine the effects of training on clinical and biomechanical outcomes, Mann-Whitney tests compared pre- and post-training data. Non-parametric statistical tests were used as has been recommended for single-subject motor control and biomechanical studies (based on the understanding that variability does not only exist across individuals, but also within individual subjects) (Kratochwill, 1978; Bates and Bates, 1996). Viable foot strikes (range: 3–6) served to provide the within-subject observations for each biomechanical outcome for each assessment timepoint. Alpha was set at 0.05.

In addition to the statistical analyses, to examine the clinical meaningfulness of the changes in walking speed, observed changes were compared to minimal clinically important difference (MCID) values for the 10mWT, which range from small (0.05 m/s) to substantial change (0.10–0.16 m/s) (Perera et al., 2006; Tilson et al., 2010; Barthuly et al., 2012). An MCID threshold of 34.4 m was used for the 6MWT (Tang et al., 2012). Because MCIDs have not been established for the biomechanical measures, to examine the meaningfulness of the biomechanical changes, when a statistically significant difference was observed, we also compared the magnitude of the observed difference to reported minimum detectable change (MDC) thresholds—i.e., peak paretic propulsion 1.8 %bw, peak paretic braking 2.5 %bw (Campanini and Merlo, 2009), stride length 11.96 cm (Geiger et al., 2019), and cadence 8.58 steps/min (Geiger et al., 2019). All data are reported using median and interquartile range (IQR). Data organization, visualization, and statistical analyses were performed using custom MATLAB scripts (Mathworks, Natick, MA, USA).

## Results

### Feasibility

The study participant tolerated all aspects of REAL training without reportable adverse events. Notably, the participant tolerated wearing the close-conforming interface of the exosuit on the paretic leg over an extended amount of time during high-intensity walking practice without skin irritation or other comfort issues, which are important biocompatibility considerations in soft robotics (Cianchetti et al., 2018). The daily *Speed*_*Target*_ was achieved for all completed walking bouts, indicating the approach used to compute *Speed*_*Target*_ was reasonable. The participant completed all five planned training sessions and 19 out of 25 (i.e., 76%) training bouts. The six missed bouts (i.e., 1 treadmill and five overground bouts) were not completed due to minor technical issues related to the exosuit's battery connection and Bluetooth communication. None of these technical issues resulted in walking disturbance and were uneventfully resolved. The participant was able to actively engage with the physical therapist in setting gait pattern goals related to paretic propulsion and was able to self-assess his performance to build on and refine these goals over subsequent training bouts. At the exit interview, the participant noted that a daily frequency may present logistical constraints on scheduling but denied cumulative fatigue as a concern.

### Efficacy Based on Clinical Outcomes

#### Primary: Comfortable Walking Speed

**Figure 3:**
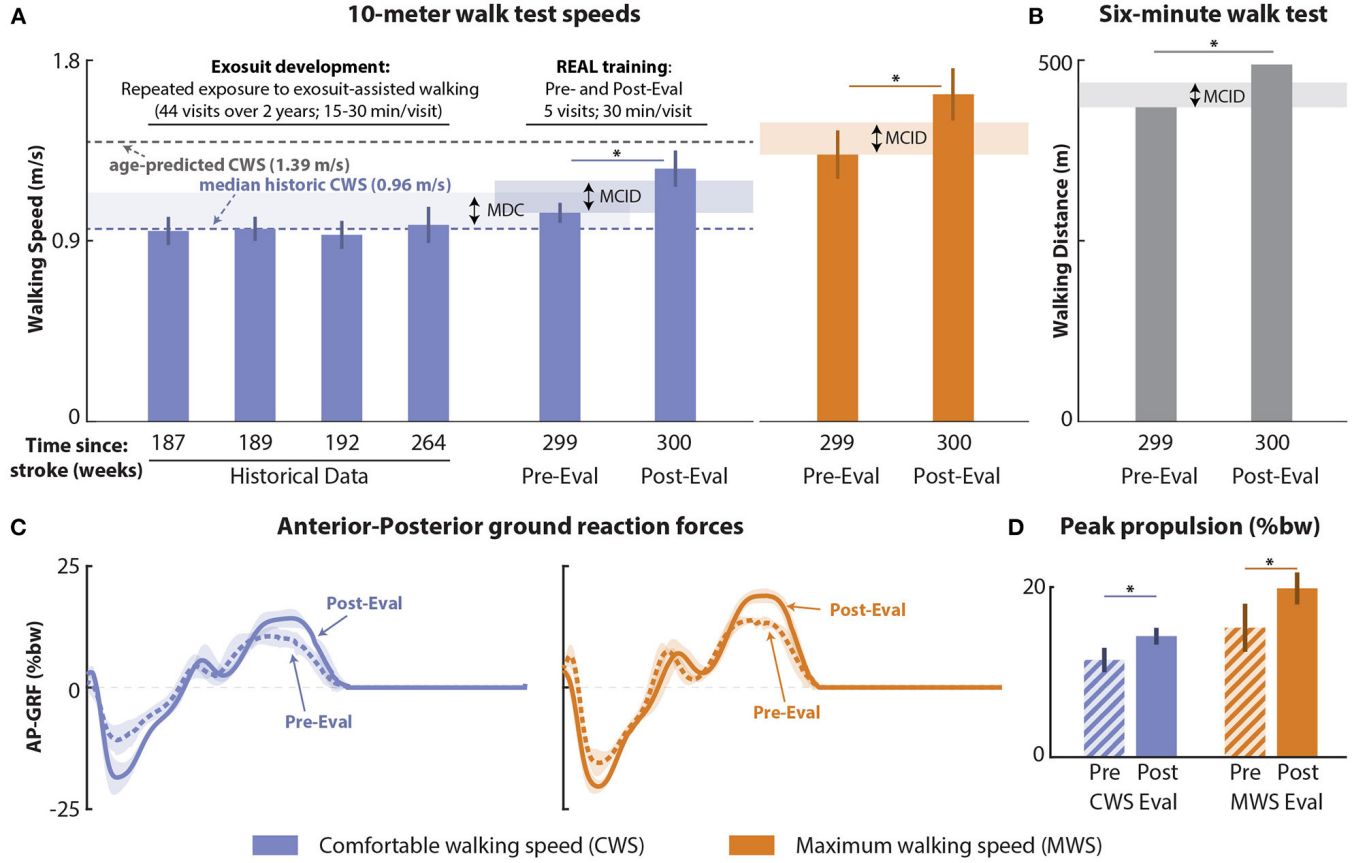
Effects of Robotic Exosuit Augmented Locomotion (REAL) training on 10-m walk test speeds and 6-minute walk test distance. **(A)** Five comfortable walking speed measurements repeatedly assessed over 2 years prior to the onset of REAL training (four historical timepoints and one pre-training evaluation conducted immediately before the training period), post-training evaluation comfortable walking speed, and pre-to-post maximum walking speeds. **(B)** Pre-to-post comparisons of 6-minute walk test distance. **(C)** Time series of anteroposterior ground reaction forces (AP-GRF) of each speed condition; **(D)** Peak propulsion of each speed condition. CWS, Comfortable walking speed; MWS, Maximum walking speed; MDC, Minimum detectable change; MCID, Minimal clinically importance difference; *: (*p* < 0.05).

The baseline assessments of 10mWT CWS that were repeated over a 2-year period were all within the MCID (Perera et al., 2006; Tilson et al., 2010; Barthuly et al., 2012) and statistically stable (*p* > 0.05). Baseline CWS had a median (IQR) of 0.96 (0.06) (Figure 3A). After REAL training, a clinically meaningful (Perera et al., 2006; Tilson et al., 2010; Barthuly et al., 2012) increase of 0.30 m/s was observed.

#### Secondary: Maximum Walking Speed and 6-Minute Walk Test Distance

Similarly, the 10mWT MWS at pre-training was 1.33 (0.12) m/s and, after REAL training, increased by 0.30 m/s. Moreover, 6MWT distance at pre-training was 435 m and, after REAL training, increased by 59 m. These improvements in MWS and 6MWT distance (Figures 3A,B) were clinically meaningful, exceeding known MCIDs (Perera et al., 2006; Tilson et al., 2010; Barthuly et al., 2012; Tang et al., 2012).

### Efficacy Based on Biomechanical Outcomes

#### Propulsion Biomechanics

Improvements in propulsion biomechanics complemented the improvements in CWS and MWS (Figures 3C,D, Table 1). Peak paretic propulsion significantly increased by 2.8 %bw at CWS and by 4.63 %bw at MWS (*p's* < 0.05), both of which exceeded the known MDCs (Campanini and Merlo, 2009). Similarly, paretic propulsion impulse increased by 0.58 %bw·s at CWS and by 0.84 %bw·s at MWS (*p's* < 0.05). Consequently, interlimb propulsion symmetry increased for both the propulsion peak (Δ: CWS: +5.12%, MWS: +6.08%) and propulsion impulse (Δ: CWS: +5.97%, MWS: +2.09%).

**Table 1:** Biomechanical effects of REAL training.

**Measure**	**Comfortable walking speed**			**Maximum walking speed**		
	**Pre**	**Post**	**Sig**.	**Pre**	**Post**	**Sig**.
**Global speed metric:**						
^†^Walking speed (m/s)	1.04 (0.05)	1.26 (0.09)	0.04^>^	1.33 (0.12)	1.63 (0.13)	0.02^>^
**P Spatiotemporal metric:**						
Cadence (steps/min)	101.3 (5.8)	110.6 (4.3)	0.04^>^	107.6 (3.8)	123.1 (3.5)	0.02^>^
^†^Stride length (m)	1.22 (0.10)	1.37 (0.04)	0.04^>^	1.45 (0.10)	1.58 (0.07)	0.02^>^
^†^Trailing limb angle (deg)	21.51 (2.04)	27.69 (0.74)	0.04	26.80 (1.62)	31.10 (1.37)	0.02
Hip circumduction (cm)	9.90 (7.50)	6.07 (3.94)	0.04	10.33 (5.55)	7.33 (4.01)	0.52
Hip hiking (cm)	7.83 (1.03)	7.41 (1.83)	1.00	9.30 (0.75)	9.05 (0.68)	0.38
**P Propulsion and Braking forces:**						
^†^Peak propulsion (%bw)	11.43 (1.43)	14.23 (0.98)	0.04^>^	15.22 (2.82)	19.85 (1.88)	0.02^>^
^†^Peak propulsion symmetry (%)	37.52	42.64	-	37.02	43.10	-
^†^Propulsion impulse (%bw·s)	2.32 (0.35)	2.90 (0.15)	0.04	2.78 (0.26)	3.62 (0.22)	0.02
^†^Propulsion impulse symmetry (%)	43.69	49.66	-	43.10	45.19	-
Peak braking (%bw)	−11.09 (4.90)	−14.55 (3.49)	0.14^>^	−17.70 (4.89)	−20.97 (2.24)	0.52
Peak braking symmetry (%)	38.04	41.15	-	37.10	43.15	-
Braking impulse (%bw·s)	−1.23 (0.63)	−1.63 (0.24)	0.14	−2.13 (0.41)	−2.39 (0.30)	0.18
Braking impulse symmetry (%)	23.89	25.93	-	33.12	31.02	-
**P Ankle Kinetic and Spatiotemporal metrics:**						
^†^Peak ankle power (W/kg)	1.37 (0.04)	1.78 (0.13)	0.04	1.83 (0.44)	2.30 (0.64)	0.12
^†^Peak ankle moment (Nm/kg)	1.20 (0.12)	1.22 (0.17)	0.39	1.32 (0.11)	1.39 (0.18)	0.27
^†^Peak ankle angular velocity (rad/s)	6.07 (0.99)	7.02 (0.36)	0.25	6.89 (0.33)	7.82 (0.88)	0.02

*Pre- and post-training median (interquartile range) and p-values for paretic limb biomechanical parameters measured at comfortable and maximum walking speeds*.

*P, Paretic limb; Sig, Significance value (significant when p < 0.05); >: For metrics with established Minimal Detectable Change scores*,

> *symbol is included to indicate the observed change was greater than the Minimum Detectable Change; -: Not applicable*;

† *: Primary outcome measure*.

Underlying the CWS propulsion improvements were significant increases in paretic trailing limb angle by 6.18 deg and peak paretic plantarflexor power by 0.41 W/kg (*p's* < 0.05). While peak paretic ankle angular velocity increased (Δ: CWS: +15.65%), this was not significant (*p* > 0.05). For MWS, significant increases were observed for paretic trailing limb angle (Δ: 4.13 deg) and peak paretic ankle angular velocity (Δ: MWS: +13.50%) (*p*'s < 0.05), but changes in peak paretic plantarflexor power (Δ: 0.47 W/kg) were not significant. Moreover, increases in peak plantarflexor moments were observed at each speed, but were not statistically different (*p*'s > 0.05) (Table 1).

#### Secondary Biomechanical Outcomes: Spatiotemporal Parameters, Compensatory Strategies, and Braking Forces

At CWS, increases in stride length (0.15 m) and cadence (9.3 steps/min) (*p's* < 0.05) were observed and exceeded the MDCs (Geiger et al., 2019). Similar gains that exceeded the MDCs were noted during MWS, with stride length increasing by 0.13 m and cadence by 15.5 steps/min (*p's* < 0.05). Paretic limb circumduction was significantly reduced at CWS by 3.83 cm (*p* < 0.05), but not at MWS (*p* > 0.05). No significant changes in hip hiking were noted at either speed. Finally, while paretic braking force peak and impulse did not change (*p*'s > 0.05), peak braking interlimb symmetry was improved at both speeds (Δ: CWS: +3.11%, MWS: +6.05%) and braking impulse interlimb symmetry was improved at CWS (Δ: +2.04%).

## Discussion

This single-subject *consideration-of-concept* trial advances a novel gait training program designed to leverage the immediate gait benefits provided by soft robotic exosuits. More specifically, we examined the feasibility and rehabilitative potential of a high-intensity, task-specific, progressive, and individualized Robotic Exosuit Augmented Locomotion (REAL) gait training program. After 5 days of REAL training, rapid and meaningful improvements in walking speed—a highly relevant clinical target (Bohannon et al., 1988; Jarvis et al., 2019)—were observed by way of improved paretic propulsion—a critical subtask of walking commonly impaired after stroke (Bowden et al., 2006). In support of our clinical hypothesis, clinically meaningful improvements were observed in all tested outcomes. In support of our biomechanical hypothesis, improvements in propulsive biomechanics and other gait quality measures were also observed. These findings support progression from this *consideration-of-concept* trial to subsequent larger and controlled clinical trials.

### Single-Subject Research Design With Long-Term Baseline

A key consideration when interpreting the findings of this study is that the repeated baseline assessments conducted over the 2-year period before the onset of REAL training showed stability of walking speed during a time when the study participant was repeatedly exposed to our study team and to the exosuit technology. During this time period, the subject participated in 44 development-focused study visits that consisted of walking with different versions of the exosuit and in various contexts and conditions. The design of this study allows us to effectively control for the following potentially confounding factors: (i) repeated exposures to walking with the exosuit, (ii) positive social interactions between the participant and our research team, and (iii) increased familiarity with the outcome measurements. That is, simply wearing and walking in an exosuit in our research lab is not a sufficient therapeutic stimulus; the rapid and substantial locomotor improvements observed in this study appear dependent on the progressive and targeted nature of the REAL training program.

### Clinical Outcomes

In the 2 years that preceded his enrollment in this study, the study participant demonstrated a highly stable CWS of 0.96 m/s that was well-below the normative, age-predicted speed of 1.39 m/s (Bohannon, 1997; Jarvis et al., 2019). After 5 days of REAL training, a substantial 0.30 m/s increase in his CWS was observed, markedly reducing this deficit. Moreover, his MWS similarly improved by 0.30 m/s. These increases in walking speed—nearly twice the size of the established MCIDs—are encouraging given that the delivered training was a fraction of what is typical in gait interventions (Ada et al., 2003; Abbasian and Rastegar, 2018), and yet approximate (or surpass) changes induced by interventions that provide a substantially greater dose of walking practice (Bowden et al., 2013).

In our prior work, exosuits were shown to be capable of immediately increasing post-stroke walking speeds and distance (Awad et al., 2020a). We posit that the REAL training program was able to leverage these immediate effects to increase the repetition and intensity of walking practice—important parameters that can facilitate breaking through the longstanding “plateaus” in walking function observed in chronic hemiparesis (Moore et al., 2010; Lohse et al., 2014) and in this study participant. Moreover, we posit that the individualized and progressive, approach to targeting walking speed and the underlying propulsive strategy inherent to the REAL training program likely contributed to the rapid increase in walking speed observed after five REAL training sessions. Further investigation of these hypotheses in dose-response studies is warranted. As with the nature of case-based research, these responses are specific to the subject; larger samples for group-level effects are needed to extend the generalizability of the results.

### Biomechanical Outcomes

Improvement in walking speed does not differentiate post-stroke recovery from compensation (Bowden et al., 2012; Combs et al., 2012). Thus, it is necessary to examine the biomechanical changes that accompany walking speed changes to substantiate the nature of motor recovery (Bowden et al., 2012; Reinkensmeyer et al., 2016; Ardestani et al., 2019a,b; Roelker et al., 2019).

#### Propulsion

Impaired paretic propulsion is a key gait deficit underlying post-stroke walking disability (Chen et al., 2005; Bowden et al., 2008; Awad et al., 2015a; Ardestani et al., 2019b). After REAL training, significant improvements in the paretic propulsion peak, impulse, and their symmetry were observed (see Table 1, Table A1). With speed increasing while the non-paretic propulsion metrics remaining unchanged after training, the observed improvements in propulsion symmetry can be attributed to the increased generation of propulsion from the paretic limb. This finding suggests that REAL was capable of targeting the “learned non-use” deficit of the paretic limb (Alingh et al., 2021), rather than facilitating a generalized response spanning both limbs. This targeted response may be a product of the exosuit's unilateral design and the explicit instructions to the study participant to increase push-off propulsion from the paretic limb, without specific instructions to modulate propulsion from the non-paretic limb. Indeed, verbal cues have been shown to have direct effects on walking performance in individuals with chronic stroke (Parker et al., 2021), and the combination of verbal cues to increase propulsion with a propulsion-augmenting exosuit holds promise as a powerful intervention synergy.

#### Metrics Associated With Propulsion

To gain insight on the movement strategies underlying the observed propulsion improvements, the effects on trailing limb angle and plantarflexion moment—the primary determinants of propulsion (Bowden et al., 2006; Hsiao et al., 2015, 2016a,b; Lewek and Sawicki, 2019)—were evaluated. After REAL training, trailing limb angle was improved. Additionally, stride length, which is associated with trailing limb angle (Mcgrath et al., 2019) and a determinant of walking speed, significantly increased. Surprisingly, training-related changes in the plantarflexion moment were not observed. This finding aligns with other studies where healthy (Hsiao et al., 2015) and post-stroke (Higginson et al., 2006; Hsiao et al., 2016b) individuals demonstrated a preferential modulation of trailing limb angle over plantarflexion moment to modulate propulsion output. Although the participant did not generate a significantly larger plantarflexion moment, there was a substantial increase in the generation of plantarflexor power after REAL training. This may be explained by a greater increase in ankle angular velocity than ankle moment (i.e., Δ: +15.65% vs. +1.67%, respectively)—factors that contribute to ankle power. Further investigation into the neuromotor changes in plantarflexor muscle function induced by REAL training is warranted (Palmer et al., 2016; Awad et al., 2020b).

Interestingly, the increase in plantarflexor power was not observed at maximum walking speed; however, similar patterns of change were observed for ankle angular velocity (Δ +13.50%; *p* < 0.05) and ankle moment (Δ: +5.3%; *p* > 0.05). One potential explanation is that the short training period may have been sufficient to train a faster maximum walking speed, but not enough to solidify improved biomechanical profiles at that speed. A longer training duration may prove to be more effective.

#### Secondary Biomechanical Outcomes

One concern related to having individuals with post-stroke hemiparesis walk at faster speeds is the potential emergence of gait compensations and the associated negative effects on walking efficiency (Lewek et al., 2012; Awad et al., 2015b), stability, and fall risk (Vistamehr et al., 2016). However, the faster walking speeds observed after REAL training were not accompanied by increased compensations. In fact, hip circumduction was observed to be reduced at comfortable walking speed.

Opposite to propulsive forces, changes in the braking forces that act to decelerate the body during collision were also examined. In healthy steady-state walking, propulsion and braking forces must be generally equal to maintain a constant speed (Bowden et al., 2006). In post-stroke hemiparetic walking, especially for those with greater impairment, increased non-paretic propulsion is accompanied by an increase in paretic braking and reduced non-paretic braking (Turns et al., 2007). After REAL training, increases in non-paretic braking forces accompanied the increases in paretic propulsive force. In contrast, we did not observe a significant change in the paretic braking force, which corresponds with our observation of no significant change in the non-paretic propulsive force (see Table 1, Table A1). These interlimb changes reflect the coordination of propulsion and braking forces that is required during bipedal walking and contrast with the findings of other therapeutic studies where an increase in non-paretic propulsion and paretic braking compensate for persisting paretic propulsion deficits (Wakida et al., 2020).

#### Effects of REAL in Relation to Other Interventions

This single-subject study—a first step in our strategic staging of pilot studies before investing in and conducting definitive clinical trials—lacks a direct control comparison. Without a control training condition, this study cannot directly decouple the effects of REAL training from simply engaging in walking practice. While cautious interpretation is warranted, the rehabilitation potential of REAL training is evident when examining the study's outcomes relative to the collective evidence on the effects of post-stroke gait interventions on propulsion function. In brief, a recent systematic review by Alingh and colleagues (Alingh et al., 2020) reported that speed improvements were common across walking interventions, however a concomitant improvement in propulsion was evident only for interventions that specifically challenged or enabled access to the latent propulsive capacity. To our knowledge, among ankle-assisting robotic devices (Shi et al., 2019), only the Anklebot has shown therapeutic improvements in paretic propulsion following treadmill-based gait training (Forrester et al., 2016), however changes in overground walking speed were not observed at the completion of the intervention (Forrester et al., 2016). Conversely, a training program combining fast treadmill training with functional electrical stimulation (FES) to the ankle muscles demonstrated improvements in both paretic propulsion and walking speed (Awad et al., 2014). FES, however, has limitations related to rapid muscle fatigability and the reduced neuromuscular capacity of the paretic limb muscles, thus limiting the scope of suitable candidates for FES-based locomotor training (Awad et al., 2016). Exosuit-augmented gait training presents an alternative intervention to the existing state-of-the-art and may be suitable for a wider range of individuals post-stroke. Additionally, as soft exosuits are fully mobile in contrast to other systems (Forrester et al., 2016), practice across treadmill and overground conditions may enhance salience and learning transfer across environments.

Ultimately, our findings show that REAL training is capable of improving, not just walking speed, but also propulsion function, with a concurrent reduction in gait compensations. That is, REAL training may be capable of facilitating a therapeutic neuromotor response that reconciles post-stroke deficits in propulsion and walking speed, which is encouraging given that previous investigations have shown that post-stroke propulsion deficits can be difficult to address (Combs et al., 2012; Hall et al., 2012; Routson et al., 2013). Larger and controlled clinical trials are needed to establish the effectiveness and generalizability of REAL gait training compared to existing approaches (Alingh et al., 2020). An important goal for future clinical trials of the soft robotic exosuit technology is understanding the differential effects of speed-based training with and without exosuit-augmentation.

#### On Designing Future REAL Gait Training Clinical Trials

A primary objective of this study was to lay a foundation for future clinical trials of the rehabilitative potential of soft robotic exosuits. Consistent with the systematic and progressive staging of pilot studies toward larger, multicenter clinical trials as proposed by Dobkin (2009) and others (Lo, 2012), we initiated this process with a *consideration-of-concept* trial of the REAL gait training program. Next in the series is a *development-of-concept* trial that will seek to test optimized components of the REAL training program against a control comparison and to identify appropriate outcomes for future trials. In regard to outcomes, the substantial improvement in the 6MWT, which is a strong predictor of post-stroke community walking activity (Fulk et al., 2017), suggests that a potentially valuable additional outcome for future studies of REAL training is the direct measurement of community walking activity (i.e., real world steps taken per day). Additional lessons learned from this *consideration-of-concept* trial include the following: adjusting the training frequency to minimize logistical difficulties related to daily training visits, allocating additional time for familiarization to the novel training approach, implementing a formal progression algorithm that systematically defines when and how to progress REAL training, and examining the effects of longer durations of REAL training and the durability of its therapeutic effects.

### Limitations

This study has several limitations. First, the repeated baseline assessments were obtained at irregular intervals. Given the low variation in walking speeds across these time points, we believe these suitably served the purpose of establishing a stable baseline. Next, pre-to-post training changes in gait biomechanics were not assessed at matched walking speeds. Although there is wide heterogeneity among people post-stroke in the biomechanical strategy used to increase walking speed, future studies that include a speed-matched condition would allow disentanglement of changes in gait biomechanics from changes in walking speed. Also, this study was not designed to discern the rehabilitative benefits that may be unique to the gait augmentation provided by the soft robotic exosuit vs. that of speed-based gait training by itself, rather this study motivates a future controlled clinical trial.

### Conclusion

This paper describes the development of a novel gait training program augmented by soft robotic exosuit technology. This *consideration-of-concept* trial provides initial evidence that an exosuit-augmented gait training program centered on high intensity, task-specific, progressive, and individualized training elements is feasible to implement after stroke and capable of facilitating rapid and meaningful improvements in paretic propulsion, walking speed, and walking distance. This early-stage clinical investigation provides several design considerations and insights that can inform subsequent clinical trials of the soft robotic exosuit technology and next generation robot-assisted gait rehabilitation.

## Data Availability Statement

The original contributions presented in the study are included in the article/supplementary material, further inquiries can be directed to the corresponding authors.

## Ethics Statement

The studies involving human participants were reviewed and approved by the Institutional Review Boards of Harvard University and Boston University. The participant provided his written informed consent to participate in this study.

## Author Contributions

FP, TCB, DAR, and JB collected the data. FP and DAR analyzed the data. FP, TCB, DAR, JB, and LNA drafted the initial manuscript. All authors contributed to the design of the study and research questions, interpreted the results, and reviewed and approved the manuscript.

## Conflict of Interest

JB is currently an employee of Apple Inc. Apple Inc. did not play a role in this study. Patents describing the exosuit components documented in this article have been filed with the U.S. Patent Office. CJW and JB are inventors of at least one of the following patent/patent applications: U.S. 9,351,900, U.S. 14/660,704, U.S. 15/097,744, U.S. 14/893,934, PCT/US2014/068462, PCT/US2015/051107, and PCT/US2017/042286, U.S. 10,434,030, U.S. 10,843,332, U.S. 10,427,293 filed by Harvard University. CJW is a paid consultant to ReWalk Robotics. The remaining authors declare that the research was conducted in the absence of any commercial or financial relationships that could be construed as a potential conflict of interest.

## Publisher's Note

All claims expressed in this article are solely those of the authors and do not necessarily represent those of their affiliated organizations, or those of the publisher, the editors and the reviewers. Any product that may be evaluated in this article, or claim that may be made by its manufacturer, is not guaranteed or endorsed by the publisher.

## Appendix

**Table A1:** Biomechanical effects of REAL training, Pre- and post-training median (interquartile range) and *p-*values for non-paretic limb biomechanical parameters at comfortable and maximum walking speeds.

**Measure**	**Comfortable walking speed**			**Maximum walking speed**		
	**Pre**	**Post**	**Sig**.	**Pre**	**Post**	**Sig**.
**NP Spatiotemporal metrics:**						
^†^Stride length (m)	1.03 (0.03)	1.33 (0.09)	0.012^>^	1.38 (0.15)	1.56 (0.11)	0.02^>^
^†^Trailing limb angle (deg)	20.58 (0.99)	24.50 (2.74)	0.04	26.32 (2.19)	28.83 (2.74)	0.02
**NP Propulsion and Braking metrics:**						
^†^Peak propulsion (%bw)	19.03 (1.42)	19.14 (3.47)	0.69	25.89 (3.46)	26.21 (2.36)	0.38
^†^Propulsion impulse (%bw·s)	2.99 (0.49)	2.94 (0.59)	0.84	3.67 (0.40)	4.39 (1.14)	0.17
Peak braking (%bw)	–23.83 (2.52)	–31.83 (7.97)	0.03^>^	–29.04 (2.65)	–39.72 (4.98)	0.02^>^
Braking impulse (%bw·s)	–3.91 (0.54)	–4.65 (0.47)	0.01	–4.30 (0.30)	–5.30 (0.32)	0.02
**NP Ankle metrics:**						
^†^Peak ankle power (W/kg)	3.14 (0.70)	2.94 (0.23)	1.00	3.93 (0.63)	3.74 (0.22)	1.00
^†^Peak ankle moment (Nm/kg)	1.71 (0.10)	1.66 (0.12)	0.06	1.80 (0.05)	1.83 (0.20)	0.26
^†^Peak ankle angular velocity (rad/s)	5.08 (0.64)	5.71 (0.21)	0.10	6.08 (1.18)	6.06 (0.68)	1.00

*NP, Non-paretic limb; Sig, Significance value (significant when p < 0.05); >: Where MDC are available*,

> *symbol is included to signify greater than the Minimum Detectable Change*;

† *: Primary outcome measure*.
